# Biomolecular Interaction Analysis of Gestrinone-anti-Gestrinone Using Arrays of High Aspect Ratio SU-8 Nanopillars

**DOI:** 10.3390/bios2030291

**Published:** 2012-08-14

**Authors:** Francisco J. Ortega, María-José Bañuls, Francisco J. Sanza, Rafael Casquel, María Fe Laguna, Miguel Holgado, David López-Romero, Carlos A. Barrios, Ángel Maquieira, Rosa Puchades

**Affiliations:** 1 Centro de Reconocimiento Molecular y Desarrollo Tecnológico, Departamento de Química, Universitat Politècnica de València, Camino de Vera s/n, Valencia 46022, Spain; E-Mails: fraorhi1@upvnet.upv.es (F.J.O.); mbpolo@upvnet.upv.es (M.-J.B.); amaquieira@qim.upv.es (A.M.); 2 Centro Láser, Universidad Politécnica de Madrid, Campus Sur, Madrid 28031, Spain; E-Mails: fj_sanza@yahoo.es (F.J.S.); rafael.casquel@upm.es (R.C.); mariafe.laguna@upm.es (M.F.L.); m.holgado@upm.es (M.H.); 3 Instituto de Sistemas Optoelectrónicos y Microtecnología, Universidad Politécnica de Madrid, ETSI de Telecomunicación, Ciudad Universitaria s/n, Madrid 28040, Spain; E-Mails: dlromero@die.upm.es (D.L.-R.); carlos.angulo.barrios@upm.es (C.A.B.)

**Keywords:** micro-nanofabrication, SU-8, optical interrogation, label-free nanobiosensing, gestrinone, immunoassay affinity constant determination

## Abstract

In this paper, label-free biosensing for antibody screening by periodic lattices of high-aspect ratio SU-8 nano-pillars (BICELLs) is presented. As a demonstration, the determination of anti-gestrinone antibodies from whole rabbit serum is carried out, and for the first time, the dissociation constant (K_D_ = 6 nM) of antigen-antibody recognition process is calculated using this sensing system. After gestrinone antigen immobilization on the BICELLs, the immunorecognition was performed. The cells were interrogated vertically by using micron spot size Fourier transform visible and IR spectrometry (FT-VIS-IR), and the dip wavenumber shift was monitored. The biosensing assay exhibited good reproducibility and sensitivity (LOD = 0.75 ng/mL).

## 1. Introduction

The development of powerful analytical tools has undergone a significant advance in the last years. Nevertheless, new improvements capable of ensuring affordable, rapid, reproducible, and sensitive analyses in a cost-effective way are mandatory for a broad range of application fields, such as healthcare, food safety, defense, environment or drug control. Among biosensing techniques, immunoassays are widely used for diagnostic testing and monitoring. They are based on the specific affinity reaction of antibodies to antigens. Generally, excellent values of sensitivity, specificity, and short reaction times, which are essential factors, are achieved by this methodology [1]. Detection based on immunoassay is used in many different devices and technologies such as reactive strips, ELISA or flow cytometry [1].

Immunoassays can be classified according to different criteria. As for the type of detection, one can distinguish between immunoassays requiring external markers to detect binding (labeled immunoassays) and those that do not need these markers (label-free immunoassays). The first ones show several drawbacks such as preparation of tracers, limited flexibility, and additional development steps. Label-free immunoassays display advantages such as cost-effectiveness, short analysis time, and operational simplicity. Interestingly, in many optical label-free biosensors, the biological reaction can be detected by a refractive index change.

Remarkable results have been accomplished through optical label-free biosensors [2,3], highlighting surface plasmon resonance (SPR) [4,5,6], porous silicon [7,8] and slot-waveguide resonators [9] based biosensors, Mach-Zehnder interferometers [10,11], directional couplers [12], and micro-ring [13] and disk [14] resonators.

Recently, we demonstrated label-free biosensing by means of SU-8 nano-pillars arrays [15], which were named BICELLs (Biophotonic sensing cells). The model system of bovine serum albumin/anti-bovine serum albumin (BSA/anti-BSA) was employed to demonstrate this concept. BICELLs’ structures consist of a periodic lattice of high-aspect ratio of SU-8 nano-pillars where the immobilization of BSA protein and the later specific recognition by anti-BSA antibodies were monitored. A limit of detection (LOD) of 2.3 ng/mL was achieved for aBSA recognition. At the same time, the biofilm thickness coating (BSA-aBSA complex) on the sensing surface of the arrays was estimated.

For the construction of BICELLs, SU-8 was selected because of its fluidic properties [16], performance and compatibility with micro-nano-processing [17], high refractive index for sensing purposes [18], and capability for direct adsorption of biomolecules [19]. Also, the device can be interrogated vertically, which avoids the use of complex coupling systems and packaging, thus providing cost-effectiveness. The vertical optical interrogation of these nano-structures can be done both by reflection employing SiO_2_ as substrate, or by transmission, with ITO-based substrates [20]. Furthermore, an improvement of the sensitivity with these nano-pillars in comparison with a single SU-8 layer of similar thickness of the pillar height has already been demonstrated [15].

Label-free detection techniques have been used for the determination of target analytes in serum, with no further treatment. For example, levels of anti-BSA in serum have been measured by scattering biophotonic microarray imaging of BSA-modified gold nanoparticles [21], with a sensitivity of 250 ng/mL. On the other hand, for the specific determination of target species in solution, the knowledge of the immunoassay kinetic parameters, such as the dissociation constant K_D_, is very important [22].

In this paper we describe the label-free biosensing of anti-Gestrinone antibodies from rabbit blood serum and, for the first time, the BICELLs are used to determine the affinity constant of the immunorecognition process. Also, the affinity constant for the system BSA/anti-BSA is obtained and compared with already existing data in order to check the quality of performance of the measurement platform. Thus, the utility of the BICELLs to perform reliable estimations of the dissociation constants working in heterogeneous format is demonstrated.

## 2. Experimental Section

### 2.1. Reagents

SU-8 2000.5 and SU-8 developer were provided by MicroChem Corp. (Newton, MA, USA). Ethanolamine and sulfuric acid 95–98% were purchased from Sigma-Aldrich (Madrid, Spain).

The buffers employed were: PBS 1× (10 mmol/L sodium phosphate, 137 mmol/L NaCl, 2.7 mmol/L KCl, pH 7.4) and PBS-T (PBS containing 0.05% Tween 20).

The gestrinone hapten-Horseradish peroxidase conjugate (HRP-h-G) and the anti-gestrinone antiserum from rabbit were synthesized as previously described [23].

Albumin chicken egg (OVA), Streptavidin-Atto 655, Gold-labeled goat anti-rabbit antibody (GAR-Au), 3,3',5,5'-tetramethylbenzidine (TMB) liquid substrate from membranes, and silver enhancer solutions A and B were purchased from Sigma-Aldrich (Madrid, Spain). Cy5-labeled goat anti-rabbit antibody (GAR-Cy5) was provided by GE Healthcare (Uppsala, Sweden).

### 2.2. Experimental Techniques

SU-8 layers were deposited on SiO_2_ substrates with a spin-coater; model WS-400BX-6MPP/LITE from Laurell Technologies Corp. (North Wales, PA, USA). The hot-plate model Agimatic E-C was from JP-Selecta (Barcelona, Spain). 2D periodical patterns of e-beam single-shots were performed on the SU-8 layers by using a CRESTEC CABL 9500 High Resolution electron-beam lithography system at 50 keV e-beam energy. BICELLs were optically characterized through a Fourier transform visible and infrared Bruker Vertex 70 spectrometer. To analyze a single BICELL, a Bruker Hyperion 1000 microscope with an optical condenser that provides an interrogation angle of an incidence from 15° to 22° and a magnification of 15×, was employed. Also, a confocal microscope Leica DCM3D was used to check the integrity of the structures.

Microarray fluorescence measurements were obtained with a homemade surface fluorescence reader (SFR) equipped with a highly sensitive CCD camera [24].

### 2.3. Experimental Procedures

#### 2.3.1. Nanofabrication of BICELLs

SU-8 nano-structures were manufactured by selective e-beam exposition of certain regions followed by chemical development, as reported recently [25]. BICELLs consisted of 60 × 60 µm^2^ of rhombic lattices of SU-8 high-aspect ratio nano-pillars with a pitch of 800 nm. The nano-pillar size was around 200 nm in diameter and 420 nm in height over a 1 µm thick SiO_2_ layer on a silicon substrate.

#### 2.3.2. Immobilization and Bioavailability Studies on Planar Surfaces

For the aforementioned immobilization studies, glass slides were spin-coated with 1 mL of SU-8, baked and cured according to the supplier fabrication specifications.

Different concentrations of GAR-Cy5 and streptavidin-Atto 655 (from 0 to 50 μg/mL) in PBS 1× were printed on the surface as 50 nL drops with a non-contact arrayer, creating a matrix of 1 × 3 spots for each concentration. The fluorescence intensity of the spots was registered using the SFR to obtain a standard curve for the probes. After incubating for 2 h at room temperature in a dark, humid chamber, planar chips were washed first 5 min with PBS-T, then 5 min with water and finally air-dried. The fluorescence was registered with the SFR at 635 nm. The signal intensity of the spots was quantified, and the amount of immobilized bioreceptor calculated from the respective calibration curve.

Following the procedure described above, HRP-h-G dilutions at 1, 5, 10 and 20 μg/mL in PBS 1× were immobilized on planar SU-8 chips creating a matrix of 1 × 4 spots for each concentration. After incubating for 2 h at room temperature in a humid chamber, each chip was washed with both PBS-T and water, and then dried. Twenty microliters of 1% OVA or 0.1 M ethanolamine in PBS were deposited on the surface and spread under a coverslip. After incubating for 30 min at room temperature, the chip was washed 5 min with PBS-T, water and air-dried. Rabbit anti-gestrinone serum at different dilutions from 1/10,000 to 1/200 in PBS-T was extended over the chip using a cover slip, and incubated at room temperature for 10 min. After washing with PBS-T and water, the chip was treated with 20 μL of GAR-Au 1/200 in PBS-T for 10 min, washed with PBS-T, then with water and dried. Finally, the microarray was developed using silver enhancer solution for 10 min, until the dark precipitate appeared in those spots showing a positive biorecognition assay.

#### 2.3.3. HRP-h-G Conjugate Immobilization and Anti-Gestrinone Antibody Recognition on the BICELLs

After fabrication, the BICELLs were treated by immersion with sulfuric acid 95–98% for 10 s followed by a washing with deionized water (DI)-H_2_O at room temperature and dried with N_2_. After that, the BICELLs were checked by confocal microscopy.

Several HRP-h-G conjugate solutions at different concentrations ranging from 0 µg/mL to 100 µg/mL in PBS-T were prepared. The conjugate immobilization was performed with 20 µL for each conjugate concentration for 60 min. Then, the surfaces were rinsed with (DI)-H_2_O and blown with N_2_ in a clean environment; BICELLs were optically characterized through Fourier transform visible and infrared spectrometry after each HRP-h-G incubation/washing step. The temperature was controlled at 20 ± 1 °C and the incubation was performed in a wet atmosphere. After this, 20 µL of ethanolamine 0.1 M in PBS was added to block the remaining epoxy groups on the surface and the BICELLs were washed and dried as before. Finally, 20 µL of rabbit antiserum dilution (ranging from 1/10,000 to 1/100) in PBS-T were dispensed and incubated for 60 min, then washed with (DI)-H_2_O and blown with N_2_, and optically characterized as aforementioned.

## 3. Results and Discussion

**Figure 1:**
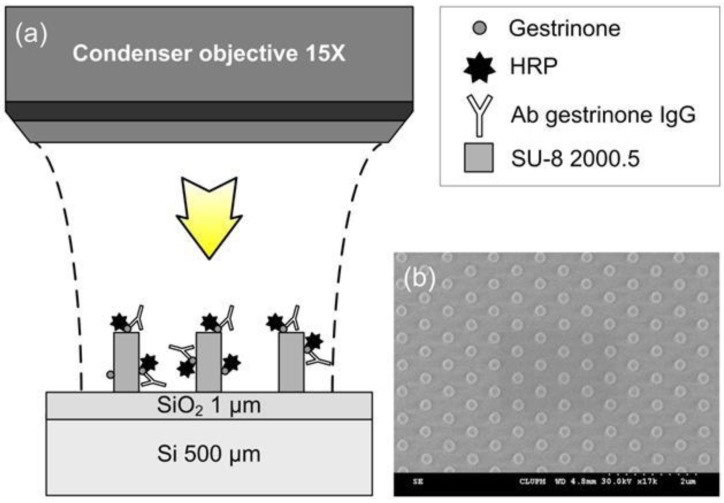
(**a**) Schematic representation of a 3D periodic array nano-pillar fabricated after the SU-8 spin coating deposition; (**b**) scanning electron microscope (SEM) micrograph of a SU-8 based biosensing cell.

The schematic representation and the 3D visualization of the BICELLs used in this work are shown in Figure 1(a,b), and it is considered to be a rhombic lattice of SU-8 nano-pillars with 200 nm in diameter, 420 nm in height and a pitch between pillars of 800 nm.

Before biosensing measurements, blank assays were carried out in order to correct the background signal from the SiO_2_ layer on the Si substrate.

**Figure 2:**
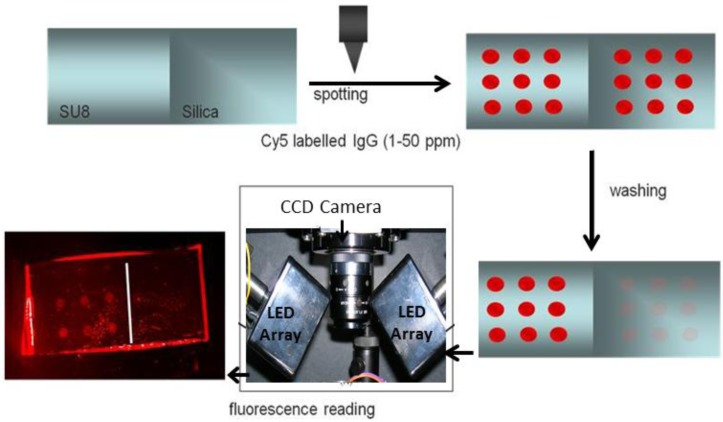
Scheme of the procedure employed to test the selective antibody immobilization on SU-8 planar chips using microarrays and Cy5 labeling.

With regard to the bioreceptor surface attachment, the epoxy groups in the SU-8 polymer allow the direct protein immobilization by the nucleophilic attack of the protein amine moieties. Thus, the first stage was to study the immobilization process. To evaluate the immobilization and biorecognition processes, planar chips consisting of glass slides coated with a layer of SU-8 photoresist were employed because our previous experience with BICELLs showed that the protocols developed in planar substrates constituted a good starting point to be applied later in the nanostructures functionalization. Microarrays (3 × 3 spots) of Atto-labeled streptavidin and Cy5-labeled goat anti-rabbit antibody were created—ranging from 0 to 50 µg/mL—on these chips with a non-contact printing automatic arrayer. Different variables such as printing buffer (carbonate buffer, phosphate buffered saline and sodium citrate saline), incubation time (from 15 min to 16 h) and washing steps were analyzed to improve the protein immobilization. The best immobilization result was obtained for 1 µg/mL of labeled probe in PBS 1× as the immobilization buffer, incubating in a humid, dark chamber for 1 h and washing by immersion in DI-H_2_O. The immobilization yield was calculated from a calibration curve, as explained in the Experimental Section, resulting a 50% of a closely packed monolayer for both the protein [26] and the antibody [27], which corresponds to a coating density of 3.3 and 1.0 pmol/cm^2^, respectively. These values were in accordance with others reported in the literature for protein and antibody immobilization on SU-8 [19,28]. Also, the selectivity of the immobilization against SiO_2_, the material creating the nanopillar platform, was evaluated. To this end, only half of the surface of the SiO_2_ planar chips was partially coated with SU-8, and an immobilization protocol was applied to both parts of the chip. After incubating and washing, the fluorescence was read (ESI, Table S1), observing protein immobilization only on the coated surface (Figure 2). Finally, the chip was washed under acidic conditions (glycine buffer, pH 2.4, 2 h), observing that the fluorescence in the microarrays decreased in less than 10% of the cases, indicating the robustness of the attachment (ESI, Table S1).

**Figure 3:**
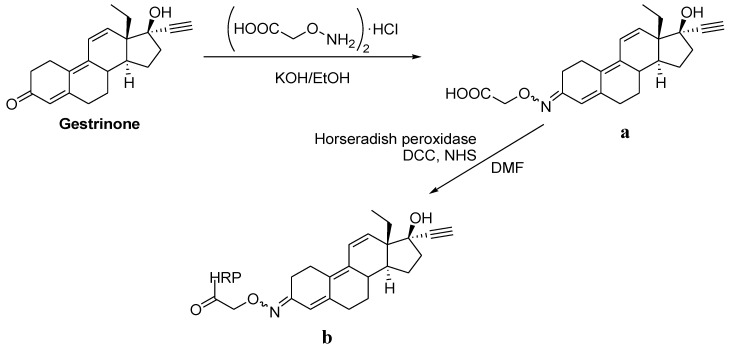
Synthesis of gestrinone oxime hapten (**a**) and gestrinone oxime hapten-horseradish peroxidase (HRP) conjugate (**b**).

Once the successful immobilization of bioreceptors on the SU-8 polymer was demonstrated, their bioavailability for a recognition event was studied. For that, the model system gestrinone/anti-gestrinone was employed. The synthetic steroid gestrinone has anti-estrogenic and anti-progesterone properties, and it is involved in several pharmacological applications including uterine pathologies [29], contraception [30,31], and endometriosis [32]. Due to gestrinone’s anabolic effects, it is the only marketed progestin included in the banned list of performance-enhancing drugs in sports (The world anti-doping code, www.wada-ama.org). In our case, gestrinone oxime was used as steroid hapten conjugated to HRP (HRP-h-G) (Figure 3), which is more appropriate to obtain specific assays because antibodies are raised against a particular moiety of a selected steroid. First of all, the immobilization of HRP was checked using the protocol used with BSA, employing TMB as enzymatic substrate for the assay development. Thus, the presence of the enzyme was detected through the appearance of a dark blue precipitate whose intensity was proportional to the amount of immobilized HRP.

**Figure 4:**
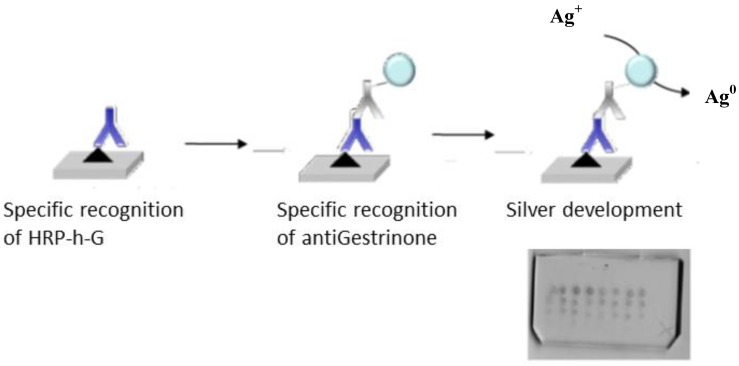
Scheme of the bioassay designed to test the bioavailability of the hapten-protein conjugate molecules attached to the SU-8 surface and scanned image obtained for the microarray.

To evaluate the bioavailability of the rabbit serum, two formats were studied in planar. For the direct format immunoassay, anti-gestrinone antibody in PBS 1× at different concentrations (1, 2 and 10 µg/mL) was immobilized on the SU-8 surface. After a blocking step with OVA or ethanolamine, HRP-h-G in PBS-T was spread out over the microarray using a coverslip and incubated for 10 min, then washed with PBS-T and water, and developed with TMB. Using this format, a positive signal was only observed for a protein-hapten conjugate concentration of 50 µg/mL. It was expected that the directly adsorbed antibody would be less efficient in reacting with the antigen that the liquid phase antibody due to denaturation and loss of its binding capacity. To test this, the indirect format was assayed (Figure 4); HRP-h-G in PBS 1× at 1, 5, 10 and 20 µg/mL was immobilized on the SU-8 planar surface accordingly to the procedure described above. Then, the surface was blocked with OVA and different dilutions of anti-gestrinone rabbit serum in PBS-T (from 1/100,000 to 1/200) were spread out, incubated for 10 min and washed. Finally, GAR-Au antibody diluted 1/200 in PBS-T was added, incubated for 10 min and washed again. Development with silver enhancer solution produced positive spots for anti-gestrinone serum dilutions up to 1/10,000, which corresponds to an anti-gestrinone antibody concentration of 100 ng/mL. The amount of specific antibody contained in the rabbit antiserum was determined from an aliquot of the serum that was purified by an affinity column and quantified by UV-vis spectrometry; the amount of specific antibody resulted in 1 mg/mL in the serum before any dilution.

Under these conditions, the hapten was recognized by the antibody with high affinity. Also, several controls avoiding the hapten or the specific antibody were done, providing negative results, which confirmed the selectivity of the interaction.

Once established the immunorecognition conditions on planar substrates, the procedure was applied to the biosensing on the BICELL nano-pillars. Thus, in a first stage, immobilization of HRP-h-G in PBS1× was monitored on the nanosensing structures. When a drop of the HRP-h-G conjugate solution was added for immobilization on the high-aspect ratio SU-8 nano-pillars, a strong solvent repulsion effect was observed due to the high hydrophobicity of the sensing material. In order to solve this, it was necessary to carry out a pretreatment with sulfuric acid to partially promote the opening of the epoxy groups, generating hydroxyl groups and increasing the surface hydrophilicity. On the other hand, the acidic opening of epoxy moieties precludes the covalent attachment of protein on the surface through their amine groups. Thus, it was necessary to reach a compromise increasing the surface hydrophilicity while preserving enough epoxy ring moieties able to perform protein covalent attachments. This was achieved with 10 s of sulfuric acid treatment. It was in agreement to other SU-8 surface hydrolysis procedures reported in the literature, where treatments for 30 min with chemical agents such as CAN (cerium ammonium nitrate) can reduce the covalent immobilization yield by around 50% [19], while other procedures like different chrome etchant [33] or PEG grafting [34] are necessary to totally avoid covalent links. Optical analysis, before and after the acidic treatment, was done using a confocal microscope to discard the degradation of the structure. The change in the surface wetability was clearly observed when adding a drop of serum dilution, as the contact angle was drastically decreased, allowing thus the drop infiltration inside the BICELLs.

In order to analyze a single BICELL, a Bruker Hyperion 1000 microscope with an optical condenser of numerical aperture of 0.4 that provides an interrogation angle of incidence from 15° to 22° and a magnification of 15× was used. Signal processing techniques were applied in order to determine the wavenumber dip position values (w) to reduce the dip position uncertainty. Thus, first the discrete Fourier transform processing (DFT) from the original signal was obtained, then the high frequency components were rejected by applying a low-pass filter (cut off number of 0.0026 cm) and finally, the inverse DFT was performed to obtain the filtered signal. The signal processing was carried out automatically, also for determining the minimum of the signal. All measured spectra were filtered with the same low-pass filter. The wavenumber shift (Δw) was monitored through the experimental spectrometry profile to establish the sensing response of the BICELL for the protein-hapten conjugate immobilization, the ethanolamine blocking and later anti-gestrinone serum recognition. The trend shown was a negative wavenumber shift as the surface biomolecule concentration increased. For each spectrum, 128 scans were carried out to reduce the signal-to-noise ratio and thus the wavenumber uncertainty u_w_.

**Figure 5:**
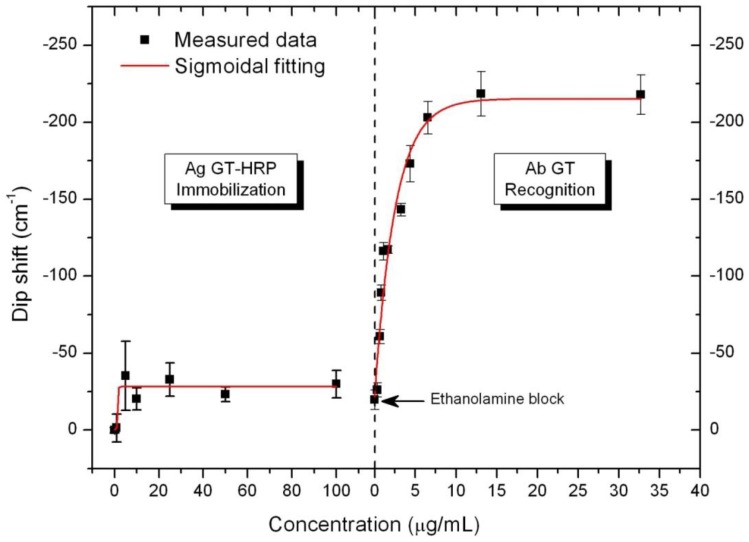
Dip shift for HRP-h-G immobilization and anti-gestrinone antibody recognition for the dip centered at 15,300 cm^−1^. Each measurement is the average of five replicates.

Figure 5 shows the dip-positions of the interference dips as a function of gestrinone hapten and anti-gestrinone increasing concentrations. It was observed that for HRP-h-G concentrations above 5 µg/mL the interference dip shift remained unchanged, indicating that the biosensing surface was saturated and the signal was due solely to immobilization of the hapten-protein conjugate. The total dip wavenumber shift (Δw) after saturation was around 30 cm^−1^. A blocking step was necessary to deactivate the remaining epoxy groups on the surface before starting the antibody addition and, as expected, the blocking step with ethanolamine 0.1 M did not yield significant signal variations.

The evaluation of the BICELLs response was carried out after incubation and washing steps to remove the unspecific binding for each concentration in static mode. The anti-gestrinone antibody recognition curve is also shown in Figure 5. A shift in the wavenumber was observed for serum dilutions ranging from 1/10,000 to 1/250, which corresponds to 0.1 and 4 µg/mL of antibody, for higher concentrations the signal remained unchanged. This suggests that anti-gestrinone antibodies can only bind to gestrinone hapten molecules available on the surface. Thus, specific recognition finishes when all the binding sites are occupied, and no surface mass accumulation is observed then.

After anti-gestrinone saturation, the total Δw was higher than the one observed for HRP-h-G immobilization. The Δw value calculated (195 cm^−1^) was found for a dip centered at 15,300 cm^−1^. The total Δw values for protein-hapten conjugate immobilization and antibody recognition were consistent with the molecular weight difference comparing the conjugate (44.2 kDa) and the antibody (150 kDa). Thus, if the gestrinone-to-protein labeling ratio was one, the expected Δw for the antibody recognition should be 3.4-fold the Δw obtained after protein conjugate immobilization. However, the labeling ratio usually is greater than one, which is consistent with obtaining a spectral shift higher than 3.4. The experimental sensitivity (m_s_) was also calculated from the interference dip wavenumber variation as a function of the antibody concentration. Thus, m_s_ resulted in 76 cm^−1^/(µg/mL) and the anti-gestrinone limit of detection (LOD) was 0.75 ng/mL, for w_res_ = 0.1 cm^−1^ (achievable in these types of interferometers). The LOD was calculated as U_w_/m_s_ where U_w_ (U_w_ = 2 u_w_) is the expanded uncertainty of a coverage probability of 95% (ESI). However, the sensitivity of the device can be improved by reducing the pitch among nanopillars, as it has already been reported [19].

Finally, the affinity constant of gestrinone-anti-gestrinone was determined using the BICELLs. Although the reaction constants calculated using the antigen or the antibody in solid-phase can be considered apparent constants rather than real immunoreaction constants, it is an interesting parameter to know when setting up a heterogeneous biosensing assay.

In the analyte-receptor recognition reaction, the dissociation constant is expressed as K_D_ = [A][R]/[AR], where [A] is the free analyte concentration, [R] is the free receptor concentration and [AR] is the analyte-receptor complex concentration. At the equilibrium, K_D_ = k_d_/k_a_, k_d_ and k_a_ are the kinetic constants for the dissociation and association process, respectively. Thus, K_D_ can be considered as the reciprocal of the analyte affinity towards the receptor (the smaller the K_D_ the higher the affinity). To calculate the dissociation constant, the receptor concentration is assumed to be [R] = [R]_total_ – [AR] and thus, when 50% of the binding sites are occupied ([AR] = 0.5[R]_total_), the dissociation constant is the free analyte concentration K_D_ = [A]. Then, using the BICELLs results for the gestrinone-anti-gestrinone system, K_D_ was the antibody concentration causing a response in the transduction equal to 50% of the total transduction change after saturation. The same determination was done with the results obtained previously for the BSA-anti-BSA system [14] to compare the obtained value with those reported in the literature [35,36,37].

**Figure 6:**
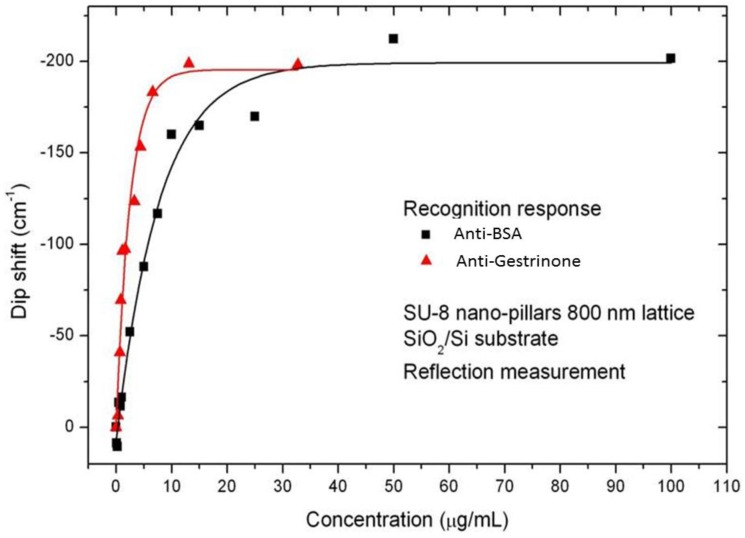
Dip shift against antibody concentration for bovine serum albumin (BSA)-anti-BSA and gestrinone-anti-gestrinone systems.

Figure 6 shows the different assay responses (BSA/ anti-BSA and gestrinone/anti-gestrinone) using the same optical transduction (SU-8 nanopillars over one micron of SiO_2_ and Si substrate). It can be seen how, when the sensing surface is saturated with antibodies, the signal level reaches a similar wavenumber displacement. In the case of anti-gestrinone, the slope was higher than for anti-BSA, and thus, the antibody concentration where 50% of the species are associated (value of K_D_) resulted higher for anti-BSA (K_D_ = 33 nM) than for anti-gestrinone (K_D_ = 6 nM). Then, the affinity was calculated as the inverse of K_D_ resulting in 0.03 nM^−1^ and 0.17 nM^−1^ for anti-BSA and anti-gestrinone, respectively. These values agree with the data reported in the literature for these systems [23,35,36,37] and demonstrate the applicability of the sensor for the determination of binding constants or biorecognition extension.

## 4. Conclusions

Highly sensitive biosensing of specific antibodies is reported in a whole rabbit serum using a cost effective method in terms of chip fabrication. This work presents for the first time the use of high-aspect ratio SU-8 nano-pillars for the calculation of antigen-antibody affinity constants, and the obtained values compare well with already existing data. The biosensing response was only due to surface modification by molecular binding during the organic films formation. The LOD of the BICELLs for anti-gestrinone molecule recognition was 0.75 ng/mL and the affinity constant was 0.17 nM^−1^. The data reported demonstrate that the platform provides useful and reliable information about the biomolecules behavior, helping to set up the best conditions to develop the biorecognition events on the label-free sensing cells. It also demonstrates that properly biofunctionalized SU-8 nano-pillars can be useful for antibody screening in heterogeneous samples such as whole serum. This can be of interest for developing sensors to detect certain diseases, where the direct measurement of the concentration of a specific antibody in blood serum with no further manipulation or purification will save time and cost.

## Appendix

The experimental sensitivity (m_s_) for the lower an-gestrinone concentrations was calculated from the interference dip wavenumber variation as a function of the analyte concentration. Thus, the best m_s_ value was obtained for dips at 15,300 cm^−1^.

In order to estimate the limit of detection (LOD), we took into account the wavenumber standard uncertainty (u_w_) during the interrogation process. This parameter can be calculated as u_w_^2^ = (w_res_)^2^/12, where w_res_ is a probability distribution with constant probability density for the interferometer wavenumber resolution. The expanded uncertainty of a coverage probability of 95% is obtained by multiplying u_w_ by a coverage factor k = 2 (U_w_ = 2u_w_), resulting U_w_ equal to 0.0577 cm^−1^ for a w_res_ = 0.1 cm^−1^, which is the maximum resolution achievable by the interferometer Vertex 70 used for the experiments. The anti-gestrinone LOD was calculated as U_w_/m_s_ resulting 0.75 ng/mL for the dip wavenumber shifts centered around 15,300 cm^−1^.

**Table S1:** Average neat fluorescence intensity (a.u.) obtained for Cy5 labelled protein microarrays immobilized on SU-8 coated and SiO_2 _surfaces after PBS washing (a) and after acidic washing (b).

Spotted protein concentration (g/mL)	SU-8		SiO_2_	
	(a)	(b)	(a)	(b)
1	n.q.	n.q.	n.q.	n.q.
10	6,140	5,902	n.q.	n.q.
25	10,050	9,654	150	n.q.
50	12,300	12,106	126	n.q.

n.q.: not quantificable.
